# Modification of the response of olfactory receptors to acetophenone by CYP1a2

**DOI:** 10.1038/s41598-017-10862-5

**Published:** 2017-08-31

**Authors:** Masashi Asakawa, Yosuke Fukutani, Aulaphan Savangsuksa, Keiich Noguchi, Hiroaki Matsunami, Masafumi Yohda

**Affiliations:** 1 Department of Biotechnology and Life Science, Tokyo University of Agriculture and Technology, 2-24-16 Naka-cho, Koganei Tokyo, 184-8588 Japan; 2 Instrumentation Analysis Center, Tokyo University of Agriculture and Technology, Koganei Tokyo, 184-8588 Japan; 3 Department of Molecular Genetics and Microbiology, Duke University Medical Center, Durham, NC 27710 USA; 4 Institute of Global Innovation Research, Tokyo University of Agriculture and Technology, Koganei Tokyo, 184-8588 Japan

## Abstract

Olfaction is mediated by the binding of odorant molecules to olfactory receptors (ORs). There are numerous proteins in the nasal mucus, and they contribute to olfaction through various mechanisms. Cytochrome P450 (CYP) family members are known to be present in the olfactory epithelium and are thought to affect olfaction by enzymatic conversion of odorant molecules. In this study, we examined the effects of CYPs on the ligand responses of ORs in heterologous cells. Among the CYPs tested, co-expression of CYP1a2 significantly affected the responses of various ORs, including MOR161-2, to acetophenone. Conversion of acetophenone to methyl salicylate was observed in the medium of CYP1a2-expressing cells. MOR161-2-expressing cells exhibited significantly greater responses to methyl salicylate than to acetophenone. Finally, we analyzed the responses of olfactory neurons expressing MOR161-2 *in vivo* using the phosphorylated ribosomal protein S6 as a marker. MOR161-2 responded to both acetophenone and methyl salicylate *in vivo*. When the olfactory mucus was washed out by the injection of PBS to mouse nasal cavity, the response of MOR161-2 to acetophenone was reduced, while that to methyl salicylate did not change. Our data suggest that CYP1a2 affects OR activation by converting acetophenone to methyl salicylate.

## Introduction

Olfaction is mediated by olfactory receptors (ORs) expressed on the cilia of olfactory sensory neurons (OSNs), which are located in the olfactory epithelium (OE)^1^. ORs bind odorant molecules and transmit the information directly to glomeruli in the primary olfactory center, the olfactory bulb (OB), and further to higher brain areas where odor perception is constructed^2^. ORs constitute the largest family in the G-protein coupled receptor (GPCR) superfamily^1^. Humans have approximately 400 OR genes, and mice have more than 1000 OR genes. Irrespective of the existence of many OR genes, each mature OSN expresses only one OR (one cell – one receptor rule)^1^. The vast majority of ORs recognize multiple odorants, and each odorant is recognized by multiple ORs. Thus, different odorants are recognized by different combinations of ORs. The olfactory system uses a combinatorial receptor-coding scheme to encode odor identities^3^.

Previous studies on the ligand selectivity of ORs were hampered by the difficulty of expressing ORs on the cell surface of heterologous cells. There were only a few reports on the identification of ligands of ORs^4, 5^. Discovery of the receptor transporting proteins (RTPs) enabled the functional characterization of ORs in HEK293T cells^6, 7^. Co-expression of the type 3 muscarinic acetylcholine receptors (M3-R) is also known to support the heterologous expression of ORs by physical interactions^8^. By the co-expression of these proteins, various ORs were functionally expressed, and their ligands were determined. It has been found, however, that for some odorants, there are differences between the ligand specificity of an odorant receptor *in vitro* and the responsiveness of its corresponding glomerulus *in vivo* ^9^. It is plausible that the odorant is converted in the nasal mucus and the metabolites activate ORs, thereby resulting in the apparent specificity differences.

There are a number of proteins in the nasal mucus, and they contribute to olfaction in various manners. Odorant-binding proteins (OBPs) are low-molecular-weight soluble proteins highly concentrated in the nasal mucus of vertebrates and in the olfactory sensilla of insects. They bind odorants or pheromones with the hydrophobic pockets. Although their physiological function has not been clearly defined, it is suggested that they contribute to olfaction by solubilizing or transporting hydrophobic odorant molecules^10^. Odorants are subjected to enzymatic conversion in mucus. Nagashima *et al*. revealed that odorants with functional groups such as aldehydes and esters are targets of metabolic enzymes secreted in the mouse mucus, resulting in their conversion to the corresponding acids and alcohols via enzymatic conversion^11^. Importantly, the enzymatic conversion of odorants in the nasal mucus appears to be fast enough to affect olfactory perception.

Various cytochrome P450 (CYP) family proteins have been known to be present in OE^12–14^. Thiebaud *et al*. investigated the *in vitro* biotransformation of odorant molecules in the rat olfactory mucus and assessed the impact of this metabolism on peripheral olfactory responses^15^. Rat olfactory mucus efficiently metabolized quinoline, coumarin and isoamyl acetate. Quinoline and coumarin are metabolized by CYPs, whereas isoamyl acetate is hydrolyzed by carboxylesterases. Metabolites of these chemicals elicited lower olfactory response amplitudes than their parent molecules. Therefore, it is reasonable to think that the enzymatic activity of CYPs expressed in OE metabolizes odorants that are dissolved in olfactory mucus and that CYP activity influences the differences in OR activities in response to odorants between *in vivo* and *in vitro* contexts. If the odorant concentration is decreased by the action of metabolic enzymes, the response of ORs should be reduced like the case in Thiebaud *et al*.^15^. Interestingly, Nagashima *et al*. reported an increase in the response of ORs, suggesting that the metabolite might induce a stronger response of ORs than the original odorant^11^.

In this study, we examined the effects of CYPs expressed in OE on the ligand response of ORs. We found that CYP1a2 affected the response of MOR161-2 to acetophenone by converting it to methyl salicylate.

## Results

### Screening of CYPs influencing odorant recognition by odorant receptors

**Figure 1:**
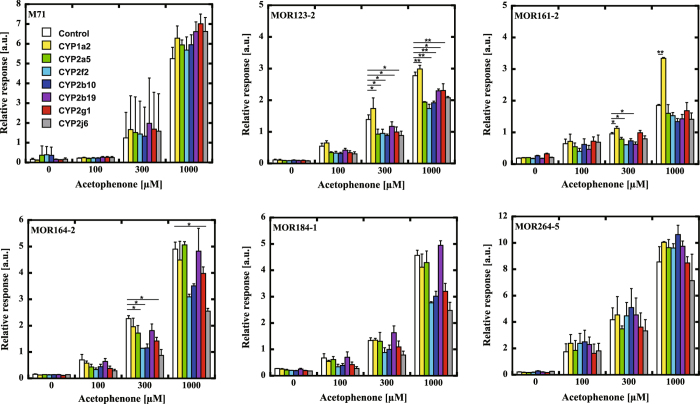
Effects of CYPs on the response of mouse ORs to acetophenone. The effects of CYPs, CYP1a2 (Yellow), CYP2a5 (Green), CYP2f2 (Light blue), CYP2b10 (Blue), CYP2b19 (Purple), CYP2g1 (Red) and CYP2j6 (Grey), on the response of ORs to acetophenone were compared with the control (without CYP, White). The ORs M71, MOR123-2, MOR161-2, MOR264-5, MOR184-1 and MOR164-2 were used for this analysis. The responses were analyzed using the Dual-Glo luciferase assay system as described in the Materials and Methods. The relative response of ORs was calculated as (Luc/RL)/(Luc/RL without odorant solution). The means ± standard deviation of three separate experiments are shown. The statistical significance was assessed using a t test (*P < 0.05, **P < 0.01).

First, we obtained cDNAs of seven CYPs, CYP1a2, CYP2a5, CYP2f2, CYP2b10, CYP2b19, CYP2g1 and CYP2j6, by PCR from a mouse OE cDNA. Then, we examined the effect of co-expression of CYP2a5, CYP1a2 and CYP2f2 on the ligand-mediated responses of mouse ORs that were known to recognize acetophenone, using a CRE-based luciferase assay in Hana3A cells (Supplementary Fig. S1)^16^. The responses of several ORs appeared to be enhanced by co-expression of CYPs. Among them, co-expression of CYP1a2 with MOR161-2 produced the significantly enhanced ligand response. Next, we examined the effects of seven CYPs on the response of MOR161-2 and compared them with those of other ORs, M71, MOR123-2, MOR264-5, MOR184-1 and MOR164-2 (Fig. 1). We observed effects of CYPs on the responses of MOR123-2, MOR161-2 and MOR164-2. However, most of them were negative effects. The most significant positive effect (p < 0.01) was observed for CYP1a2 on the response of MOR161-2. We selected the combination of CYP1a2 and MOR161-2 for further study. There was only a small increase at 300 µM, but a nearly double response at 1 mM acetophenone were observed. The effect of CYP on OR activation was observed after two steps of mass transfers, the substrate from outside to inside and the metabolic product from inside to outside. It seemed to require high concentration of substrate to observe the effect. Thus, it was difficult to obtain a linear response.

### CYP1a2 converts acetophenone to methyl salicylate in culture medium

**Figure 2:**
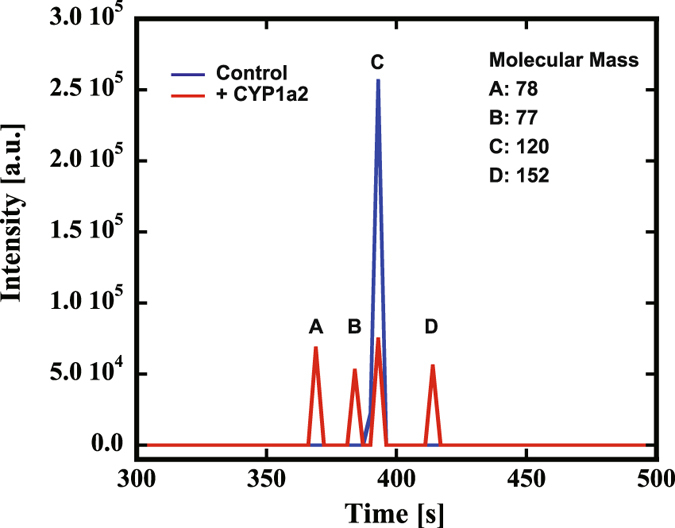
Identification of metabolites of acetophenone in the culture medium. The culture media of Hana3A cells expressing CYP1a2 (Red) and the mock transformant (Blue) were analyzed by total ion chromatography. The peaks (**A**,**B** and **D**) were analyzed by mass-spectrometry. The estimated molecular masses are shown.

Functional expression of CYP1a2 in Hana3A cells was confirmed by the P450-Glo assay using luciferin-ME as a substrate^17^. Hana3A cells expressing CYP1a2 exhibited significantly high bioluminescence compared to the mock transformant (Supplementary Fig. S2A). The bioluminescence linearly increased with the incubation time (Supplementary Fig. S2B). Next, to investigate whether CYP1a2 metabolizes acetophenone, we cultured Hana3A cells with or without expression of CYP1a2 in the culture medium containing 1 mM acetophenone, and then analyzed the culture medium by Gas Chromatography – Mass Spectrometry (GC-MS). The chromatograms of the total ion chromatography are shown in Fig. 2. Compared with the control, the peak for acetophenone (Peak C) at approximately 393 s decreased and new three peaks appeared in the culture medium of CYP1a2-expressing cells. Among the newly appearing peaks, the peak A at approximately 370 s identified to be that for DMSO with the molecular weight of 78. DMSO was used as the solvent for acetophenone, and the peak A appeared in another GC analysis of culture medium of Hana3A cells without expression of CYP1a2 (data not shown). The molecular mass of the peak B at approximately 385 s was measured to be 77. The peak B occasionally appeared in the GC analysis of culture medium of Hana3A cells without expression of CYP1a2 (data not shown) and it seems to be a chemical of the cell. Mass spectrometry analysis of the peak D at about 420 s has shown the fragment peaks with the molecular weights of 92, 120, 121 and 152. Thus, it was identified to be methyl salicylate. Because methyl salicylate was not included in CD293 medium, we concluded that acetophenone was converted to methyl salicylate by the action of CYP1a2.

### Comparison between acetophenone and methyl salicylate as odorants

**Figure 3:**
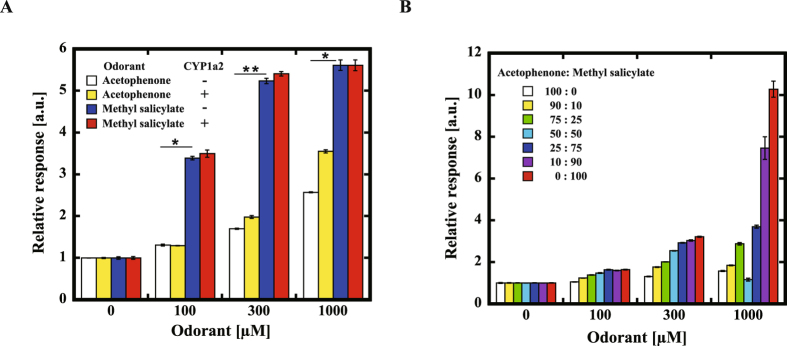
Response of MOR161-2 to acetophenone and methyl salicylate. (**A**) Comparison of the responses of MOR161-2 to acetophenone and methyl salicylate in the presence or absence of CYP1a2. The statistical significance was assessed using a t test (*P < 0.05, **P < 0.01). (**B**) Response of MOR161-2 to the mixture of acetophenone and methyl salicylate. E.g. 25:75 designates the mixture of 25% of 100 µM of acetophenone and 75% of 100 µM of methyl salicylate. The response was analyzed using the Dual-Glo luciferase assay system as described in the Materials and Methods. The relative response of ORs was calculated as (Luc/RL)/(Luc/RL without odorant solution).

**Figure 4:**
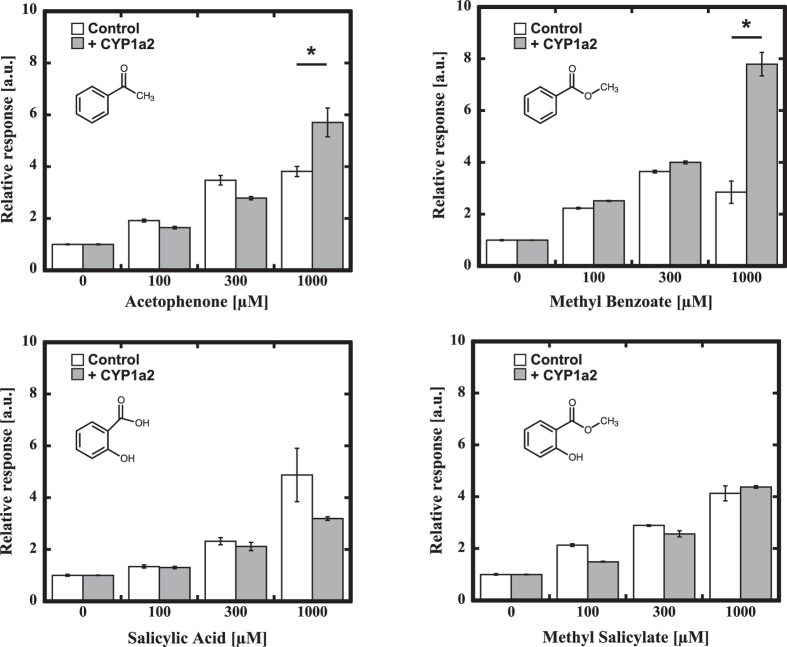
Comparison of responses of MOR161-2 to the possible metabolic intermediates, methyl benzoate and salicylate, with acetophenone and methyl salicylate. The response was analyzed using the Dual-Glo luciferase assay system as described in the Materials and Methods. The relative response of ORs was calculated as (Luc/RL)/(Luc/RL without odorant solution). The means ± standard deviation of three separate experiments are shown. The statistical significance was assessed using a t test (*P < 0.05).

We compared the response of MOR161-2 against acetophenone and methyl salicylate. MOR161-2 showed a larger response to methyl salicylate than acetophenone. Enhancement of MOR161-2 response by CYP1a2 was observed only for acetophenone (Fig. 3A). As water solubility of acetophenone is higher than that of methyl salicylate, the difference of the response of the MOR161-2 could not be attributed to the difference in solubility. To examine whether acetophenone and methyl salicylate were competing, we tested the response of MOR161-2 to the mixtures of acetophenone and methyl salicylate (Fig. 3B). The ligand-mediated response of MOR161-2 increased as the proportion of the methyl salicylate. We also tested the responses to methyl benzoate and salicylic acid, which are analogues of acetophenone and methyl salicylate. In addition to acetophenone, co-expression of CYP1a2 improved the response of MOR161-2 to methyl benzoate (p < 0.05) (Fig. 4). On the contrary, almost no effect was observed for salicylic acid or methyl salicylate. As methyl benzoate is converted to methyl salicylate by hydroxylation, it is most plausible that methyl benzoate was converted to methyl salicylate by CYP1a2.

### Effect of mucus on odorant recognition

**Figure 5:**
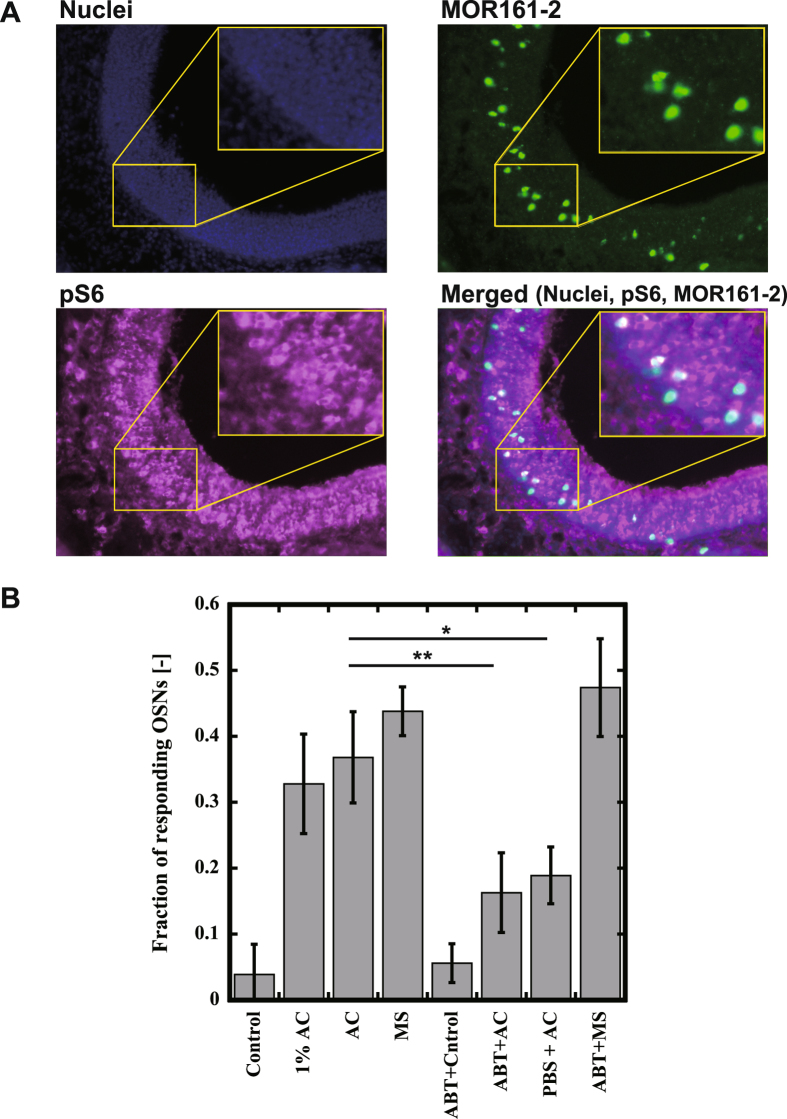
Immunofluorescence and RNA *in situ* hybridization of mouse OE for activation with acetophenone and methyl salicylate. (**A**) Mice were stimulated by a piece of filter paper spotted with 10 µL odorant and sacrificed to stain the olfactory epithelium. pS6 and MOR161-2 were detected by Immunofluorescence and RNA *in situ* hybridization, respectively. (Nuclei) nuclei of olfactory sensory neurons, (MOR161-2) RNA *in situ* hybridization for MOR161-2, (pS6) Immunofluorescence for pS6, and (Merged) Merged image of Nuclei, MOR161-2 and pS6. And then the stained sections were observed by fluorescence microscope. Images were 20× magnification. (**B**) The ratio of the responding OSNs, identified using anti-S6 antibody, to the total MOR161-2 expressing cells was calculated. The means ± standard deviation of three separate experiments are shown. The statistical significance was assessed using a t test (*P < 0.05, **P < 0.01). 1% AC, 1% acetophenone; AC, acetophenone; MS, methyl salicylate; Control, purified water without odorant. PBS, with washing nasal cavity by PBS; ABT, with washing nasal cavity by PBS containing ABT. Three mice were used for each stimulation condition. At least four sections were analyzed for each stimulation condition.

Finally, we tested whether nasal mucus affected the odor-mediated responses in OSNs expressing MOR161-2. The ribosomal S6 protein is phosphorylated in response to neuronal activation^18^. We analyzed the number of OSNs activated by a single odorant in the OE by using RNA *in situ* hybridization for MOR161-2 and *in situ* immunofluorescence analysis for phosphorylated S6 (pS6)^19^. Mice were placed in a cage with a stimulation cassette enclosing a piece of filter paper spotted with odorants (1% acetophenone, acetophenone, methyl salicylate, and water as a control). In Fig. 5A, whole cells were imaged with the fluorescence for total nuclei (Top), MOR161-2 positive cells (Middle), pS6 positive cells (Bottom). The ratios of the responding OSNs, identified using anti-S6 antibody, to the total MOR161-2 expressing cells were calculated (Fig. 5B). OSNs expressing MOR161-2 responded to 100% acetophenone was 34.7%, which was significant as that to methyl salicylate, 41.5%. Even 1% acetophenone induced the almost same level of response (34.0%) as 100% acetophenone. To evaluate the effects of nasal mucus, we washed the nose with PBS with or without CYP inhibitor, 1-Aminobenzotriazole (ABT) before odor stimulation. As ABT is small molecule with hydrophobic moiety, it can easily penetrate through the plasma membrane to inhibit CYPs in the cell. ABT inhibited the response of MOR161-2 to acetophenone (19.7%). But, it is unknown whether the effect is due to the inhibition of CYP as the almost same inhibition was observed only by PBS rinse (19.7%). On the contrary, ABT treatment did not affect the response of MOR161-2 to methyl salicylate (47.3%). Although we could not obtain the solid evidence for the effect of CYP on olfaction *in vivo*, these results indicate that some factors in the nasal mucus are responsible for the enhancement of MOR161-2 response to acetophenone.

## Discussion

The olfactory epithelium expresses various metabolic enzymes, such as cytochrome P450, dehydrogenases, oxidases, reductases, and esterases, which degrade various chemicals coming from the outside world^20–24^. The main role of the enzymes is likely to protect against infection by microorganisms or against damage by harmful chemical compounds. Previous studies have shown that xenobiotic-metabolizing enzymes including CYPs affect olfaction by metabolizing odorants^15^. The enzymes also metabolized various odorants to reduce the responses of olfactory cells. The result that the olfactory response in the nasal cavity is modified by washing the nasal cavity with Ringer’s solution further shows the effects of metabolizing enzymes on olfaction^9^.

To obtain more solid evidence for the contribution of CYPs to OR activation, we screened CYPs and ORs to select pairs of CYPs and ORs showing significant effects of CYPs. We found that CYP1a2 significantly enhanced the response of MOR161-2 to acetophenone. We observed the conversion of acetophenone to methyl salicylate in the medium of CYP1a2-expressing cells. As MOR161-2 exhibited a stronger response to methyl salicylate than to acetophenone, we concluded that the effect of CYP1a2 is due to the conversion of acetophenone to methyl salicylate. It is unclear, whether CYP1a2 catalyzes this reaction by itself, since it requires both hydroxylation and conversion of the acetyl group to a methoxycarbonyl group. It is possible that the reaction is mediated by the combination of CYP1a2 and other cellular enzymes. Potential intermediates are methyl benzoate and salicylate, and indeed MOR161-2 responded to both of them. However, in the GC-MS, we could not observe such intermediates. Further analysis will be required to delineate the full pathway of the observed odor conversion.

CYP2f2 partly decreased the response of MOR161-2 (Supplementary Fig. S1). On the contrary, the response of MOR246-2 was increased by CYP2f2 (Supplementary Fig. S1). Thus, CYP2f2 should metabolize acetophenone to a metabolite different from methyl salicylate, which increased the response of MOR246-2.

In the *in vitro* experiment, expressed CYPs should be localized in ER or mitochondria. Therefore, the event comprises of the following processes, (1) transport of the substrate into the cell, (2) conversion by CYP, (3) transport of the metabolite to the outside and (4) OR activation. Thus, it is relatively inefficient and a high concentration substrate is required to observe the effect. It is implausible that such a complicated and inefficient system functions *in vivo*. There is a report suggesting that the rat CYP may be a secreted protein in respiratory mucosa^25^. However, it is difficult to think that CYPs are secreted and function as soluble proteins outside of the cell. We think that CYPs may be exported outside of the cell as exosomes in the nasal cavity. Exosomes are biologically active vesicles secreted by different cell types including epithelial, hematopoietic, and some tumor cells. They are present in some biological fluids such as serum, urine, breast milk, and bronchoalveolar lavage fluid. As mucous fluids are rich in secreted proteins, it is reasonable that mucous fluids are also rich in exosomes. The exosomes produced by the liver and blood cells eventually secrete into the plasma, which may contain high levels of exosomes containing CYP enzymes^26^. This idea was partly supported by the fact that the response of MOR161-2 to acetophenone was significantly impaired by the washout of nasal mucous. Washout of nasal mucous decreased levels of exosomes containing CYP enzymes which could explain why the response was significantly impaired. The washout of nasal mucous also affected the responses of ORs by rinsing away other factors including OBPs. Further experiments will be required to analyze the effects of CYPs or OBPs *in vivo*.

*In vivo* experiment, 30–50% of the cells expressing MOR161-2 exhibited a pS6 signal as co-localization after stimulation by each agonist, acetophenone (34.0% for 1% AC and 34.7% for 100% AC) and methyl salicylate (41.5% for 100% MC) (Fig. 5B). It was difficult to think that the signal was not in saturation because almost no dose dependency was observed and the response of methyl salicylate was almost same as that of acetophenone. One possibility is that some portion of MOR161-2 expressing cells are immature, because RNA probe specific bound to MOR161-2 was used for staining. In the course of development, a single immature olfactory neuron cell can express multiple odorant receptor genes^27^.

Substrates in olfactory mucus are actually a mixture of various odorants. In addition, many enzymes seem to be present in olfactory mucus to metabolize odorants. To identify native ligands of ORs, we need to consider the environment around OSNs and in the olfactory mucus in the future. It appears that odorant perception by animals are constructed from unexpected complex mechanisms.

## Methods

### Chemicals

The odorant compounds were purchased from Wako Pure Chemical Industries Ltd. (Osaka, Japan) or Sigma-Aldrich Co. (St. Louis, MO). Odorant solutions were diluted to 1 M stock solutions in DMSO and kept at −20 °C until used. The CYP inhibitor ABT (1-aminobenzotriazole) was purchased from Sigma-Aldrich Co. and diluted to 400 µM in PBS.

### Plasmid construction

A synthesized DNA fragment for Rho-tag (MNGTEGPNFYVPFSNATGVVR) was subcloned into the mammalian expression vector pCI. Open reading frames for all odorant receptors were amplified from mouse genomic DNA and subcloned into pCI expression vectors containing the Rho-tag gene fragment. All CYPs were cloned from a cDNA library from mouse OE and inserted into pCI expression vectors. RTP1S and M3-R were also inserted into pCI expression vectors. The sequences of all plasmid constructs were verified by sequencing. The ORs and CYPs used in this study are listed in Supplementary Tables S1 and S2.

### Cell Culture

Hana3A cells were used to express ORs and CYPs^16^. Cells were maintained in minimum essential medium Eagle (Sigma-Aldrich Co.) containing 10% Hyclone fetal bovine serum (GE Healthcare Life Sciences, Buckinghamshire, UK) and 0.5% Antibiotic-Antimycotic Mixed Stock Solution (Nakalai Tesque, Inc., Kyoto, Japan) at 37 °C with 5% CO_2_.

### Luciferase Assay and Data Analysis

The Dual-Glo luciferase assay system (Promega, Madison, WI) was used for luciferase assays. Hana3A cells were plated on poly-d-lysine-coated 96-well plates (Thermo Fisher Scientific, Waltham, MA). Expression plasmids for ORs, RTP1S, M3-R, and CYPs were transfected into Hana3A cells using Lipofectamine 2000 (Thermo Fisher Scientific). In addition, two luciferase constructs were used, including a firefly luciferase gene driven by a cAMP-response element (CRE-Luc) and a Renilla luciferase gene driven by a constitutively active SV40 promoter (pRL-SV40). The promoter was used as an internal control for cell viability and transfection efficiency. When ORs are activated, the downstream second messenger cAMP is produced, and binding of cAMP to the cAMP-response element region leads to luciferase gene transcription and luminescence. For each 96-well plate, 10 ng of CRE-Luc, 5 ng of pRL-SV40, 5 ng of OR plasmid, 5 ng of RTP1S plasmid, 2.5 ng of M3-R plasmid, and 5 ng of CYP plasmid or the pCI empty vector were transfected. At 24 h post-transfection, the medium was replaced with 50 µl of odorant solution diluted in CD293 (Invitrogen) and incubated for 3 h at 37 °C. The activities of firefly luciferase (Luc) and Renilla luciferase (RL) were measured according to the manufacturer’s protocols. Luminescence was measured using a GloMax-Multi Detection System (Promega). Relative response of ORs was calculated as (Luc/RL)/(Luc/RL without odorant solution).

### CYP activity assay

P450-Glo CYP1A2 Induction/Inhibition Assay (Promega) was used for confirm of CYP1a2. Hana3A cells were plated on 6 well plates (Nippon Genetics) and transfected with CYP1a2 plasmid (20 or 100 ng/well) or the pCI empty vector using Lipofectamine 2000. At 24 h post-transfection, the medium was replaced with luciferin-ME as a substrate diluted in CD293 and incubated for 3 h at 37 °C. Then, the luminescence was measured.

### GC-MS analysis

Hana3A cells were plated and transfected with CYP1a2 or the pCI empty vector. At 24 h post-transfection, the medium was replaced with odorant solution diluted in CD293 and incubated for 3 h at 37 °C. Then, 500 µl of conditioned medium was collected, and organic compounds present in the medium were extracted using ethyl acetate. Gas chromatography–mass spectrometry (GC/MS) (JMS-700, Nihon Denshi) was used to examine the ethyl acetate extract. The compounds were separated using a DB-WAX column (30 m × 0.25 mm inner diameter, film thickness = 0.5 µm). The column temperature was held at 50 °C for 1.5 min, then programmed to rise to 180 °C at 25 °C/min and then to 250 °C at 10 °C/min, and finally held at 250 °C for 20 min. The interface temperature was maintained at 250 °C, and the ion source temperature was set at 200 °C. Peak identities were confirmed by matching the component mass spectra to the National Institute of Standards and Technology Mass Spectral Database and by matching the retention time and mass spectra of the peaks to the data observed for the pure compounds. To identify the products of enzymatic reaction in the nasal mucus, mass spectra were obtained in full scan mode. Quantitative analysis of products was performed by comparison with the peak area of authentic odorants in total ion chromatograms (TIC).

### Immunofluorescence and RNA *in situ* hybridization

Male and female C57BL/6 mice of 2–3 weeks old were maintained on a 12 h light-dark schedule. Each mouse was transferred to a clean disposable cage and presented with the stimulation cassette enclosing a piece of filter paper spotted with 10 µL odorant or water (control). The odorants used were acetophenone (1% (in distilled water) and 100%) and methyl salicylate. 1 h later, these mice were sacrificed, and coronal sections from the olfactory epithelium (20–25 micron thick) were cut onto slides which were stored at −80 °C. The sections were thawed and fixed in 4% PFA for 20 minutes, washed for 1 minute with 0.5% Triton X-100 in PBS and then rinsed twice in PBS. They were blocked for 1 hour in PBS containing 5% milk and 0.1% Triton X-100, and then kept overnight at 4 °C with rabbit anti-pS6 240/244 antibody at 1/400 in the blocking buffer. The sections were washed with PBS three times for 5 min each, and then incubated with Cy3-conjugated anti-rabbit antibody at 1/200 for 1 h at 4 °C.

To visualize nuclei, sections were washed with PBS two times for 5 min each followed by incubation with 1/10000 of 1% bisbenzimide in PBS for 5 min at room temperature.

Expression of MOR161-2 was analyzed by RNA *in situ* hybridization. The probe was prepared by the addition of the T3 start site to the 3′ end of the pCI plasmid primer followed by incorporation of digoxigenin (DIG) using T3 RNA polymerase (Promega) and alkaline degradation to get labeled probes of around 200 bp. Slides were prepared as described above and fixed in 4% PFA for 15 minutes and washed twice in PBS. They were acetylated in 1.2% triethanol amine and dropwise addition of acetic acid. They were washed in PBS for 5 minutes and prehybridized in buffer for an hour in large plates soaked in 5XSSC and 50% formamide at 58 °C. 1 µL of the aforementioned labeled probe in 200 µL of the prehybridization buffer was pipetted on to the slides and covered with a layer of parafilm and kept overnight at 58 °C. The slides were thoroughly washed in 5XSSC and then in 0.2XSSC twice for 30 minutes, 5 minutes in PBS and then blocked for an hour. Then, anti-digoxigenin-POD antibody solution was added for 45 min. The detection was performed by tyramide signal amplification (TSA) technology. Sections were washed in 0.1% BSA-PBS followed by the TSA stock solution FITC (1/400) in PBS containing 0.003% H_2_O_2_ for 10 min at room temperature. 1-Aminobenzotriazole (ABT), an inhibitor of cytochrome P450 enzymes, was used to evaluate the effect of CYP1a2 on the response of MOR161-2 in the mouse nose. PBS containing 400 µM ABT was injected into the mouse nasal cavity before stimulation with odorants.

The ratio of the responding OSNs, identified using anti-S6 antibody, to the total MOR161-2 expressing cells was calculated. All images were captured on Zeiss Axioskop 2 fluorescent microscope using Q capture pro. Images were then analyzed using ImageJ. Three mice were used for each stimulation condition. At least four sections were used for each condition. The animal procedures performed in this study were conducted at Duke University Medical Center with the approval by the Animal Care Committee of Duke University Medical Center. All animals were used under Duke University Medical Center Animal Care institutional guidelines.

## Electronic supplementary material

Supplementary information
