# Supplementary material for: Modification of the response of olfactory receptors to acetophenone by CYP1a2
Source: Sci Rep. 2017 Aug 31;7:10167. doi: 10.1038/s41598-017-10862-5 (PMC5579037; doi:10.1038/s41598-017-10862-5)
Supplement: Supplementary file 1 — Supplementary information [file 41598_2017_10862_MOESM1_ESM.pdf]

Modification of the response of olfactory receptors to acetophenone by CYP1a2

Masashi Asakawa[1], Yosuke Fukutani[1], Aulaphan Savangsuksa[1], Keiich Noguchi[2], Hiroaki Matsunami[3, 4], Masafumi Yohda[1,4] *

[1] Department of Biotechnology and Life Science, Tokyo University of Agriculture and Technology, 2-24-16 Naka-cho, Koganei, Tokyo 184-8588, Japan.

[2] Instrumentation Analysis Center, Tokyo University of Agriculture and Technology, Koganei, Tokyo 184-8588, Japan.

[3] Department of Molecular Genetics and Microbiology, Duke University Medical Center, Durham, NC, 27710, USA

[4] Institute of Global Innovation Research, Tokyo University of Agriculture and Technology, Koganei, Tokyo 184-8588, Japan.

*Corresponding author:
Department of Biotechnology and Life Science, Graduate School of Technology, Tokyo University of Agriculture and Technology, Koganei, Tokyo 184-8588, Japan
yohda@cc.tuat.ac.jp

Supplementary Fig. S1 Screening of mouse ORs that are affected by co-expression of CYPs.

ORs that are known to respond to acetophenone were used for screening. Relative responses of cells transfected with odorant receptor and CYPs, CYP2a5 (Green), CYP1a2 (Yellow), CYP2f2 (Light blue) or an empty vector (White). The relative response of ORs was calculated as (Luc/RL)/(Luc/RL without odorant solution). The means ± standard deviation of two separate experiments are shown.

Supplementary Fig. S2 Confirmation of functional expression of CYP1a2 in Hana3A cells.

CYP1a2 activity in the cell free extract of Hana3A cell was analyzed using the P450-GloTM assay with luciferin-ME as a substrate. Bioluminescence of Hana3A cells expressing CYP1a2 was compared with that of the mock transformants with pCI empty vector. (A) Dose response of CYP1a2 plasmid and control plasmid (pCI). Bioluminescence was measured after 3h incubation. (B) Time course of bioluminescence of Hana3A cells transfected with 20ng of CYP1a2 plasmid.

Supplementary Fig. S3 Control experiments for immunofluorescence and RNA in situ hybridization of mouse OE.

Mice were mock stimulated by a piece of filter paper spotted with 10 μL water. pS6 and MOR161-2 were detected by Immunofluorescence and RNA in situ hybridization, respectively. (Nuclei) nuclei of olfactory sensory neurons, (MOR161-2) RNA in situ hybridization for MOR161-2, (pS6) Immunofluorescence for pS6, and (Merged) Merged image of Nuclei, MOR161-2 and pS6. And then the stained sections were observed by fluorescence microscope. Images were 20x magnification.

Supplementary Table S1 The list of ORs used in this study.

All odorant receptor open reading frames were amplified from mouse genomic DNA and subcloned into pCI expression vectors containing a Rho-tag.

Supplementary Table S2 The list of CYPs used in this study.

All CYPs were cloned from a cDNA library from mouse OE and inserted into pCI expression vectors.

Supplementary Fig. S1

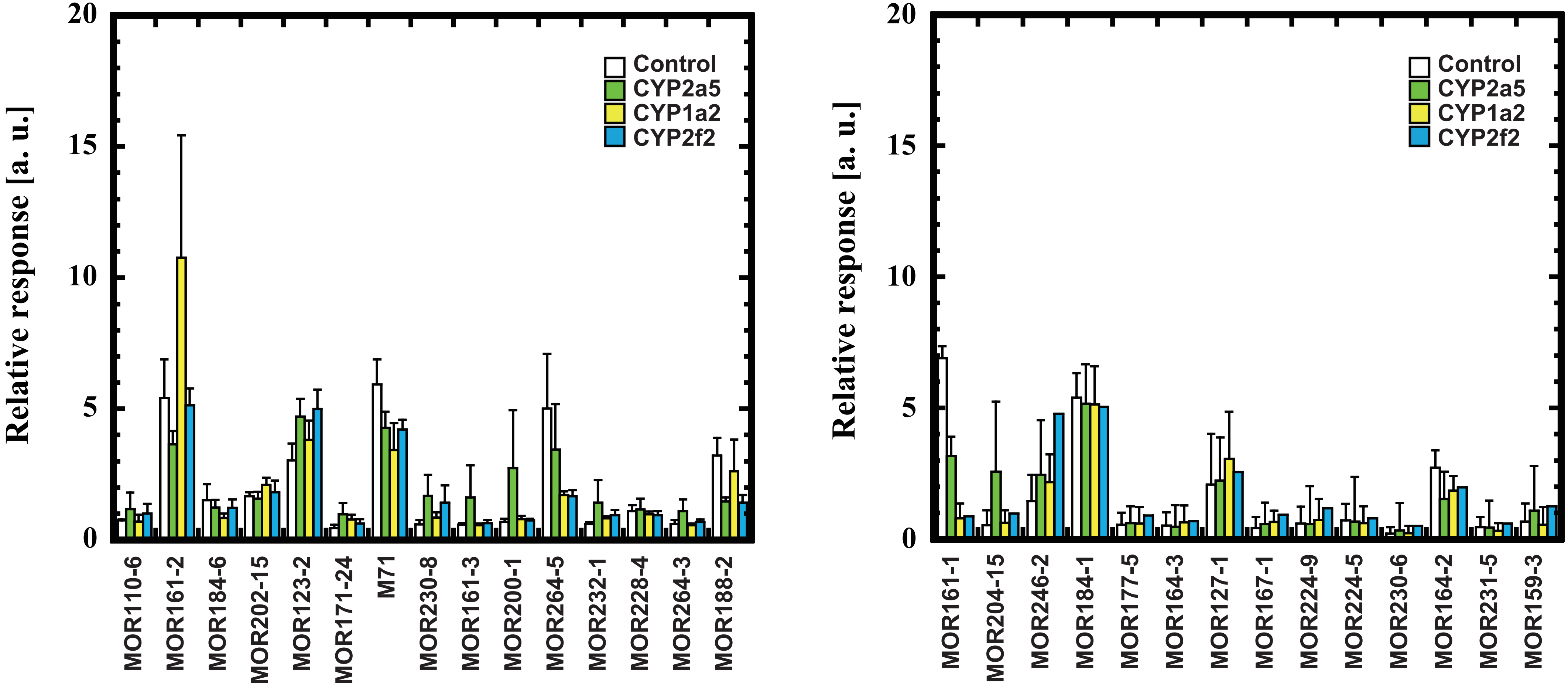

Supplementary Fig. S2

(A)

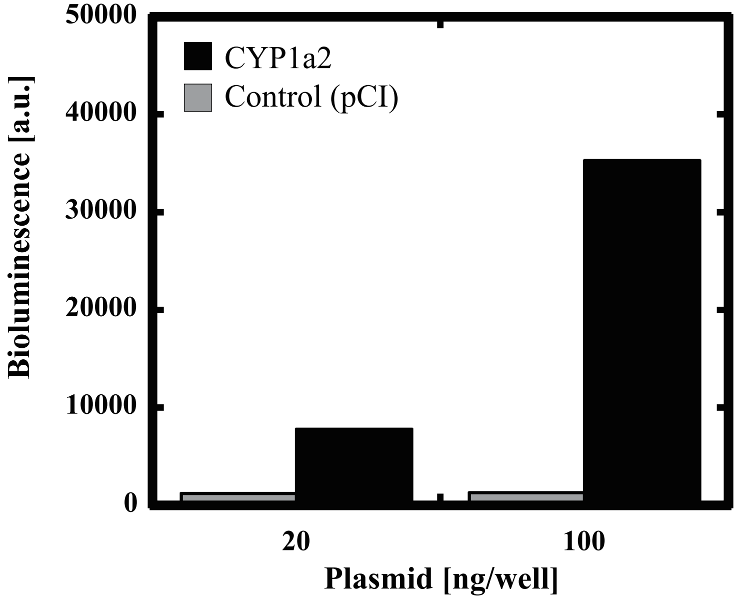

(B)

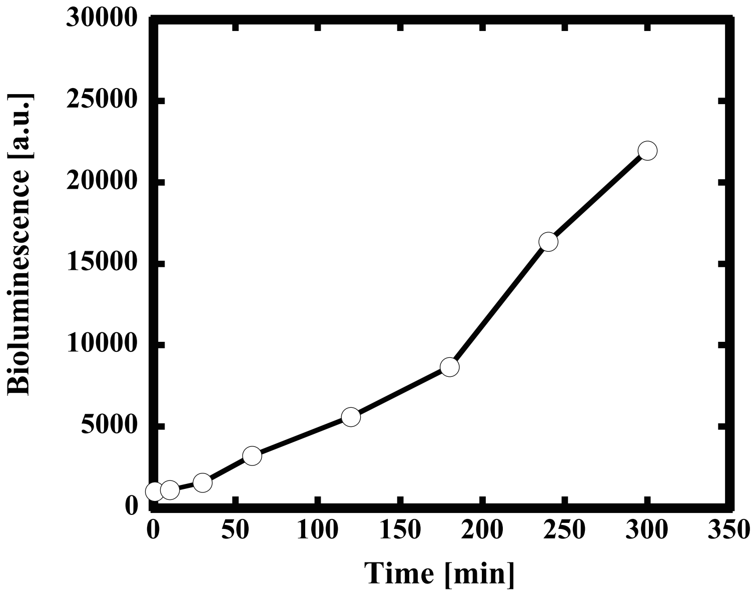

Supplementary Fig. S3

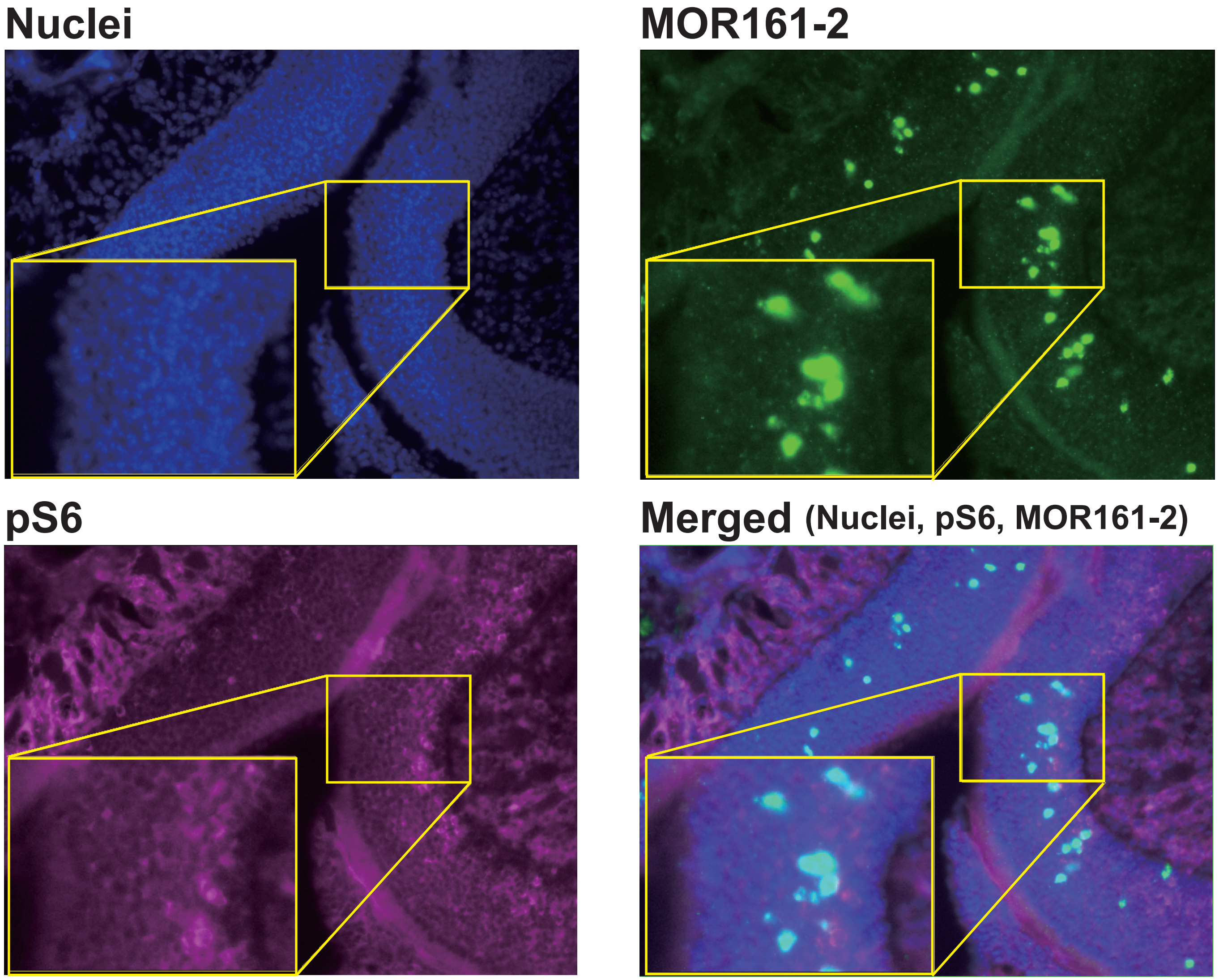

Supplementary Table S1

The list of ORs used in this study

NAME	ORDB	ACCESSION
MOR110-6	Olfr811	NM_146552.1
MOR161-2	Olfr874	NM_146882.2
MOR184-6	Olfr178	NM_146997.2
MOR202-15	Olfr1467	NM_146691.1
MOR123-2	Olfr432	NM_146716.2
MOR171-24	Olfr937	NM_146439.1
M71	Olfr151	NM_207664.2
MOR230-8	Olfr1199	NM_146458.1
MOR161-3	Olfr922	NM_146781.2
MOR200-1	Olfr1031	NM_001011759.2
MOR264-5	Olfr1126	NM_146837.2
MOR232-1	Olfr1257	NM_146982.1
MOR228-4	Olfr1274	AY318479.1
MOR264-3	Olfr1124	NM_147028.2
MOR188-2	Olfr1054	AY318250.1
MOR161-1	Olfr876	NM_146883.2
MOR204-15	Olfr488	NM_146732.1
MOR246-2	Olfr727	NM_146319.2
MOR184-1	Olfr174	AY317259.1
MOR177-5	Olfr153	NM_206823.1
MOR164-3	Olfr918	NM_146375.2
MOR127-1	Olfr1496	NM_146989.2
MOR167-1	Olfr905	NM_146804.2
MOR224-9	Olfr958	NM_146330.1
MOR224-5	Olfr961	NM_146504.1
MOR230-6	Olfr1183	NM_146529.2
MOR164-2	Olfr923	NM_146816.2
MOR231-5	Olfr1242	NM_146968.2
MOR159-3	Olfr361	NM_146368.1
MOR211-4P	Olfr1505	NM_001011850.1

Supplementary Table S2

NAME	ACCESSION
CYP1a2	NM_009993.3
CYP2a5	NM_007812.4
CYP2f2	NN_007817.2
CYP2b10	NM_009999.3
CYP2b19	NM_007814.1
CYP2g1	NM_013809.1
CYP2j6	NM_010008.4
